# Protective Effects of Linearthin and Other Chalcone Derivatives from *Aspalathus linearis* (Rooibos) against UVB Induced Oxidative Stress and Toxicity in Human Skin Cells

**DOI:** 10.3390/plants10091936

**Published:** 2021-09-17

**Authors:** Akeem O. Akinfenwa, Naeem S. Abdul, Jeanine L. Marnewick, Ahmed A. Hussein

**Affiliations:** 1 Department of Chemistry, Cape Peninsula University of Technology, Symphony Rd., Bellville 7535, South Africa; oa.akeemlaja@gmail.com; 2 Applied Microbial and Health Biotechnology Institute, Cape Peninsula University of Technology, Symphony Rd., Bellville 7535, South Africa; sheikabduln@cput.ac.za (N.S.A.); marnewickj@cput.ac.za (J.L.M.)

**Keywords:** skin cells, UV light-exposure, *Aspalathus linearis*, linearthin, aspalathin derivatives, isolation, characterization

## Abstract

Skin cells suffer continuous damage from chronic exposure to ultraviolet light (UV) that may result in UV-induced oxidative stress and skin thinning. This has necessitated the formulation of cosmeceutical products rich in natural antioxidants and free radical scavengers. *Aspalathus linearis* (rooibos) is an endemic South African fynbos plant growing naturally in the Western Cape region. The plant is rich in phenolics and other bioactives with a wide spectrum of health benefits. The chemical study of an acetonic extract of green *A. linearis* afforded a novel compound named linearthin (**1**) and two known dihydrochalcones, aspalathin (**2**) and nothofagin (**3**). The chemical structure of the novel compound was elucidated based on spectroscopic data analysis. The bio-evaluation of the isolated chalcones in vitro for protection against UVB-induced oxidative stress were systematically assessed by examining cell viability, metabolic activity, apoptosis, and cytotoxicity using HaCaT and SK-MEL-1 skin cells models. It was observed that pre-treatment with tested samples for 4- and 24 h at low concentrations were sufficient to protect skin cells from UVB-induced damage in vitro as evidenced by higher cell viability and improved metabolic activity in both keratinocytes (HaCaT) and melanocytes (SK-MEL-1). The results further show that the pre-treatment regimen employed by this study involved some degree of cellular adaptation as evidenced by higher levels of reduced glutathione with a concomitant decrease in lipid peroxidation and lowered caspase 3 activity. Furthermore, compound **1** was most cytoprotective against UVB irradiation of HaCaT cell line (over 24 h) with an IC_50_ of 282 µg/mL and SK-MEL-1 cell line with IC_50_ values of 248.3 and 142.6 µg/mL over 4 and 24 h, respectively. On the other hand, HaCaT cells exposed to **2** over 4 h before UVB irradiation showed the highest degree of cytoprotection with an IC_50_ of 398.9 µg/mL among the four studied samples. These results show that linearthin (**1**) and the two glycoside dihydrochalcone of *A. linearis* have the potential to be further developed as antioxidant cosmeceutical ingredients that may protect skin against UVB-induced damage.

## 1. Introduction

The human skin is continuously exposed to environmental stressors, among these, solar UV radiation is considered the most ubiquitous, damaging and causing skin immune suppression [1]. The UVB component of solar radiation is strongly linked to its potential to generate excessive reactive oxygen species (ROS) that damage cellular macromolecules and is recognised as an initiator of photocarcinogenesis and mutagenesis [2]. Although the skin is equipped with an intricate defence system, encompassing both enzymatic and non-enzymatic components to protect it from these adverse biological effects, excessive exposure to UV radiation can overwhelm and diminish these systems [3,4]. Studies have shown that excessive ROS production and/or its ineffective elimination is implicated in triggering pro-inflammatory and apoptotic signalling cascades that are implicated in many cutaneous pathological processes [2,5].

With a notion to protect humans against the adverse effects of UV radiation, there is growing interest in the use of naturally occurring plant products, including polyphenols, for the prevention of UV-induced skin photodamage, with ethnobotanical antioxidants showing considerable promise [6,7,8]. *Aspalathus linearis* (Brum.f) Dahlg. (Fabaceae) (rooibos) is a fynbos shrub endemic to the Cape Floristic Region of South Africa [9]. It is widely consumed globally as a herbal tisane but is increasingly being used as an additive in skincare products [10,11,12,13]. *A. linearis* commonly exists as a green (unfermented) rooibos with a chemical profile that reflects high chalcone contents including the unique chalcone, aspalathin (ASP). The red type (fermented rooibos) has been shown to result from the oxidative/enzymatic fermentation of the green rooibos plant material under certain conditions. The oxidation reactions that occur during the fermentation process change the chemical profile of the plant and showed a decrease in the dihydrochalcone derivative content, especially aspalathin [14,15]. Hence, green rooibos is a better option for targeted isolation of aspalathin and polyphenolic compounds reported as active ingredients for cosmeceutical products [16,17]. Extracts prepared from green rooibos possess potent antioxidant, anti-inflammatory and anti-tumour properties [18,19,20,21] with evidence supporting its photoprotective effects in the skin [12,20,22]. These effects are strongly linked to its unique phytochemical composition and bioactive compounds [23].

ASP derivatives are known to have a dihydrochalcone skeleton and are connected with a *C*-glycosidic linkage [11,24]. Several studies have demonstrated the potent antioxidant properties of ASP [24,25,26] with percutaneous permeation studies revealing some degree of absorption through the skin [27] which supports its use in cosmeceutical preparations. However, evidence on the role of ASP in UVB-induced skin damage is still lacking.

This study was designed to assess the possible interactions between a green rooibos total extract and three isolated compounds with chalcone-related structures and UVB radiation using two different human cell lines, i.e., HaCaT, an immortalized human keratinocyte cell line derived from non-tumorigenic epidermal cells representing the pre-malignant state and SK-Mel-1, a melanoma cell line established from patient-derived tumour cells. We examined the potential of the extract and test compounds/samples to attenuate UVB-induced oxidative stress and maintain cell viability in both cell lines.

## 2. Results

### 2.1. Characterization of the Isolated Compounds

**Figure 1:**
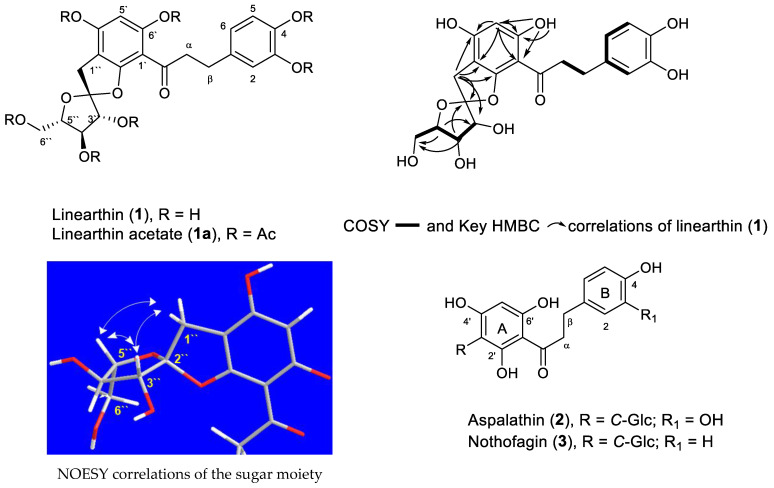
Chemical structure of the isolated compounds and COSY, HMBC and NOESY correlations of compound **1**.

Chromatographic purification of an acetone extract of *A. linearis* using different techniques including semi-prep HPLC yielded three pure chalcone derivatives, isolated and identified as linearthin (**1**), aspalathin (**2**) and nothofagin (**3**), respectively (Figure 1).

Compounds **2** and **3**, consist of *C*-glycosides linked to dihydrochalcone structures and their presence in rooibos is reported herein for the first time. Compound **3** was previously isolated from *Nothofagus fusca* and *Schoepfia chinensis* [27,28,29]. Compounds **2** and **3** were identified as aspalathin and nothofagin by comparison with literature data (Table 1) [30,31]. In addition to compounds **2** and **3**, we report in this study the isolation and full characterisation of compound **1** for the first time.

Compound **1** (10 mg) was isolated as an amorphous white-yellowish powder. The HRMS of compound **1** showed a peak at *m*/*z* 443.1135 [M-H]^+^ corresponding to the molecular formula C_21_H_22_O_10_. The UV showed absorption at 332 nm and its FTIR spectrum exhibited bands at 1705 and 3340 cm^−1^ indicating carbonyl and hydroxyl functional groups. Its NMR spectra (Table 1) demonstrated dihydrochalcone skeleton features with a 3,4-disubstituted ring B. ^1^H-NMR showed a singlet at 5.82 (H-5′), aromatic signals of 1,3,4-trisubstituted benzene ring at 6.49 (H-6); 6.63 (H-5); 6.61 (H-2) and two methylene groups, at 3.18/3.35 (CH_2_-α); 2.75 (CH_2_-β) in addition to 1-deoxysugar signals at 3.10/2.91 (CH_2_-1″); 3.88 (H-3″); 3.83 (H-4″); 3.77 (H-5″) and 3.43/3.54 (H-6″). The CH-2″ protons showed a large geminal coupling (*d*, *J* = 16.4 Hz) that indicated the group existed in a cyclic structure and was isolated by two fully substituted carbons on both sides.

**Figure 2:**
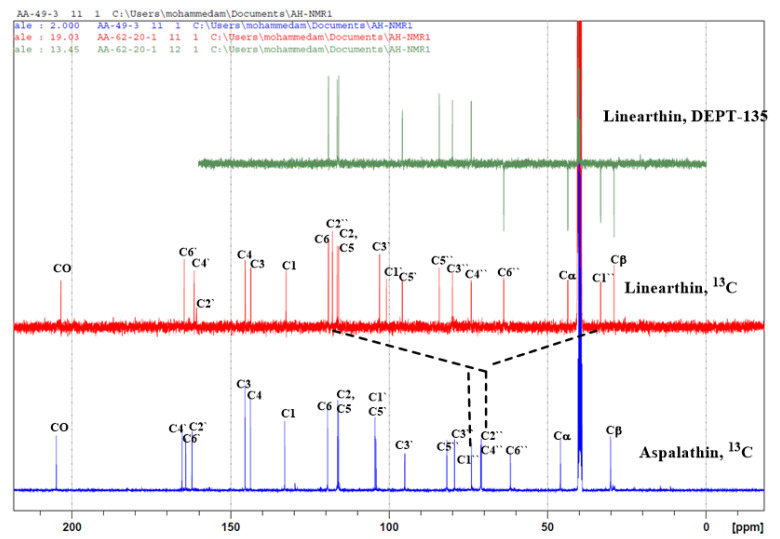
Comparisons of carbon-13 and DEPT-135 between linearthin (**1**) and aspalathin (**2**).

The ^13^C, and DEPT-135 (Figure 2) showed 21 carbons, 15 of them at δ_C_: 132.4 (C-1); 116.3 (C-2); 143.6 (C-3); 145.4 (C-4); 115.9 (C-5); 119.1 (C-6); 100.7 (C-1′); 161.2 (C-2′); 103.0 (C-3′); 161.4 (C-4′); 95.9 (C-5′); 164.6 (C-6′); 43.6 (C-α); 29.1 (C-β) and 203.3 of a carbonyl group belonging to a dihydrochalcone skeleton. The other six carbons at δ_C_: 33.3 (C-1″); 117.8 (C-2″); 80.0 (C-3″); 74. 1 (C-4″); 84.2 (C-5″); 63.8 (C-6″) belong to a deoxy sugar. The chemical shift of C-2″ (117.8) indicated a dioxygenated carbon. The COSY spectrum of the heptaacetate derivative **1a** showed correlations between H3″/H4″; H4″/H5″; H5″/H6″ which support the location of the dioxygenated carbon at the C2″ position. The above NMR data are very similar to that of compound **2**, especially regarding rings A and B and the three carbon unit between the two rings. Differences exist only in the signals of the sugar moiety. Table 1 shows the presence of a CH_2_ group (δ_C_ 33.3) and a dioxygenated carbon (117.8) in the new structure which indicated a deoxymonosaccharide (Figure 2). According to the molecular formula as determined by HRMS (C_21_H_22_O_10_) and when compared to aspalathin (C_21_H_23_O_11_) this indicates the presence of extra unsaturation, which should be a cyclic ether. The acetate derivative (linearthin heptaacetate, **1a**) of compound **1** showed the expected seven acetate groups which further supports the presence of the extra cyclic ether.

The 2D NMR spectra fully established the structure of compound **1**, in particular, the HMBC correlations spectrum (Figure 1) which showed cross-peaks between H-5′/C-1′, C-3′, C-6′, C-4′; H-1″/C-2′, C-3′, C-4′, C-2″, C-3″; H-2″/C-4″; H-4″/C-2″, C-6″; H-5″/C-3″, C-6″; 5′-OH/C-1′, C-2′, C3′ among others and confirmed the structure as shown in Figure 1. This compound was given the trivial name linearthin. The relative configuration of the sugar moiety was established from ^1^H-^1^H couplings and NOESY spectra of the heptaacetate derivative **1a**. The spectra from the original compound **1** could not be interpreted because of the complexity of the sugar proton area. The NOESY spectrum of the heptaacetate derivative showed a typical fructofuranoside configuration and showed correlations between H-1″, H-3″ and H-5″; and confirmed the *syn*-relationship and the spiral linkage at C-2″, while H-4″ showed a correlation with H-6″. Attempts to crystalize the acetate derivative to establish the absolute configuration of the isolated compounds were not successful. To the best of our knowledge and according to the SciFinder database, linearthin (**1**) has not been previously isolated before from a natural source, and it represents a very rare skeleton type containing a *C*-fructofuranoside unit.

### 2.2. Cytoprotective Effect of the Tested Samples on Skin Cells Exposed to UVB Irradiation

HaCaT and SK-MEL-1 cells were pre-treated with total extract and tested samples (TE, compounds **1**, **2**, and **3**) for 4 or 24 h before exposure to UVB irradiation. The MTT assay was then used to determine the cytoprotective effects of the tested samples.

After exposure to UVB the viability of HaCaT cells not pre-treated with tested samples decreased to 72% and 63% for the 4- and 24-h experiments, respectively, when compared to cells not exposed to UVB or tested samples, while the viability of SK-MEL-1 was found to have decreased to 77% and 70% for the 4- and 24-h experiments, respectively when compared to cells not exposed to UVB or tested samples (data not shown).

**Figure 3:**
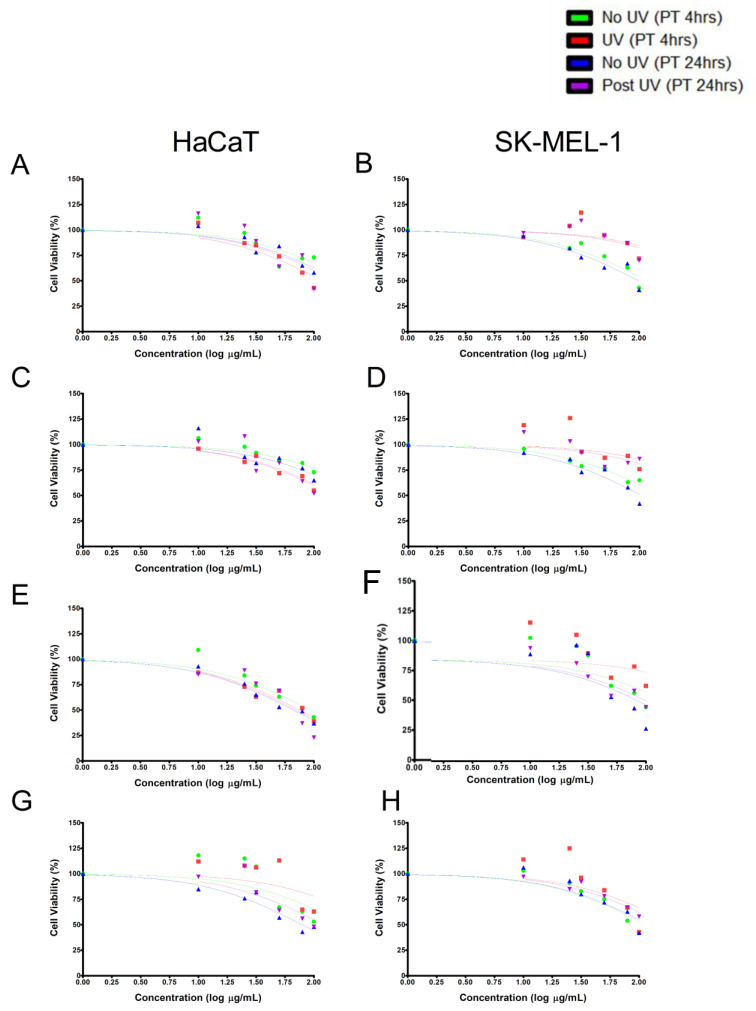
Effect of the tested samples pre-exposure [4- and 24-h; TE (**A**,**B**); compound **1** (**C**,**D**); compound **2** (**E**,**F**); compound **3** (**G**,**H**)] on UVB irradiation in HaCaT and SK-MEL-1 cells. The MTT cytotoxicity curve alluded to time and dose-dependent cytotoxic effects by the tested samples at higher concentrations.

Following the observation that the UVB model was cytotoxic, the two cell lines were incubated with the tested samples (0–100 µg/mL) for 4- and 24-h. The results showed that all the tested samples showed time and dose-dependent cytotoxic effects against the treated cell lines independent of UVB exposure. It was interesting to note that at lower concentrations the tested samples were able to stimulate cell metabolic activity and cell viability (Figure 3A–H) in UVB and non-UVB exposed cells. These increases in cell viability indicate a reduction in UVB-induced cytotoxicity. Also noted is the SK-MEL-1 melanocytes showed greater resilience to UVB-induced toxicity when compared to HaCaT and that these protective effects were potentiated by pretreatment with the tested samples.

### 2.3. Determination of the IC_50_ Values of the Tested Samples under UVB Exposure

**Figure 4:**
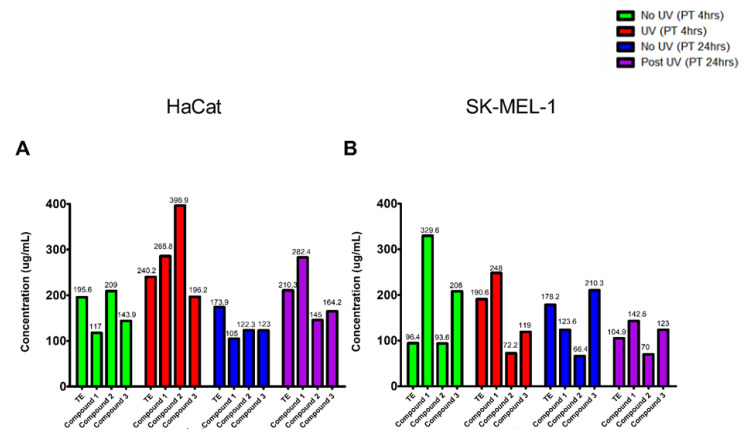
The IC_50_ concentrations of the tested samples on the two cell lines, HaCaT (**A**) and SK-MEL-1 (**B**), after 4- and 24-h pre-exposure to determine cytoprotection against UVB.

To confirm which of the tested samples were the least cytotoxic after UVB irradiation, the half-maximal inhibitory concentration (IC_50_) values examining cell viability as a measure of metabolic outputs were determined. Results confirmed that HaCaT cells exposed to compound **2** for 4 h before UVB irradiation showed the highest degree of cytoprotection among the four studied extracts, with an IC_50_ of 398.9 µg/mL while 24-h pre-exposure to **1** was shown to be the most effective isolate against UVB irradiation with an IC_50_ of 282 µg/mL. In the SK-MEL-1 cell line, we observed compound **1** to be the most effective isolate in protecting cells against UVB irradiation with IC_50_ values of 248.3 and 142.6 µg/mL over 4 and 24 h, respectively. The IC_50_ values reported in Figure 4, are defined as the concentration of isolate required for 50% inhibition of viable cell number in vitro, obtained from each cytotoxicity curve (Figure 3), these reflect the differences where a higher IC_50_ value is attributed to a higher concentration of isolate being needed to reduce cell viability. Based on this data we selected concentrations of tested samples to be used for all subsequent assays.

### 2.4. Effect of the Tested Samples on Cell Viability

**Figure 5:**
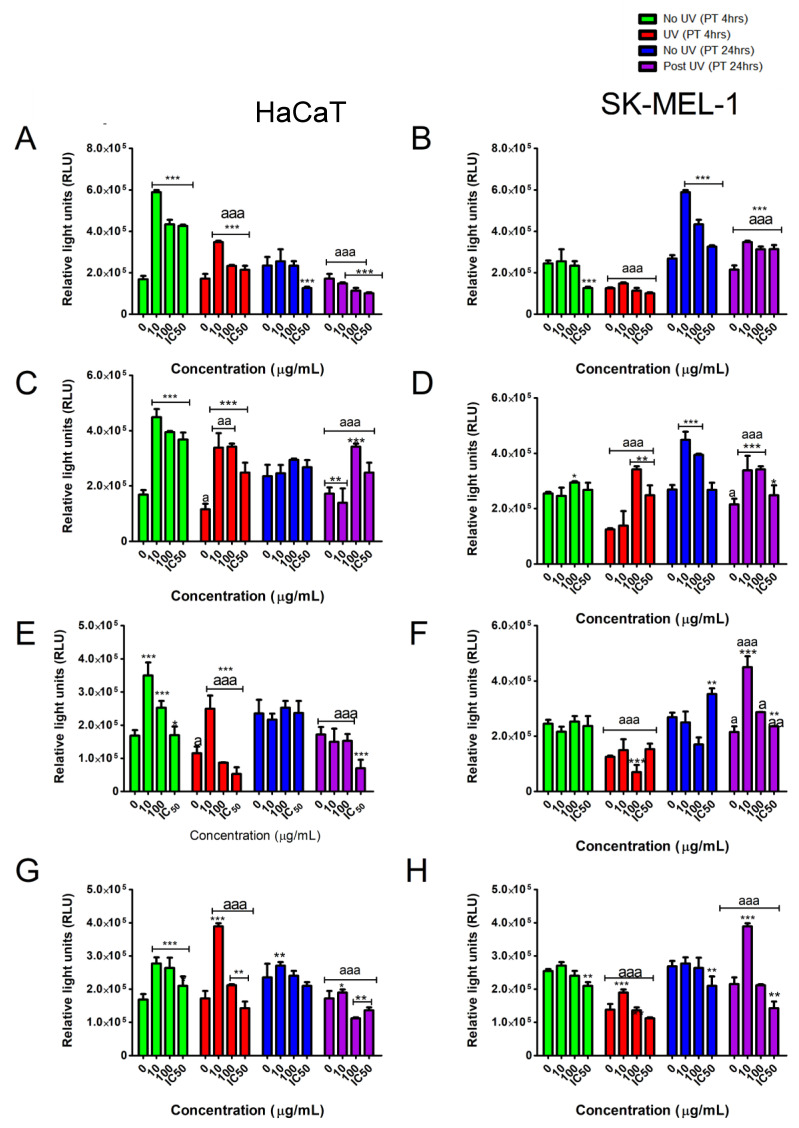
Cells were treated with selected concentrations of the tested samples [10, 100 and the IC_50_ (µg/mL); TE (**A**,**B**); compound **1** (**C**,**D**); compound **2** (**E**,**F**); compound **3** (**G**,**H**)] before exposure to UVB and the levels of ATP were detected using luminometry. Vertical lines indicate standard deviation. * *p* < 0.05, ** *p* < 0.01, *** *p* < 0.001 control vs. experimental concentrations; ^a^ *p* < 0.1, ^aa^ *p* < 0.01, ^aaa^ *p* < 0.001 No UVB irradiation vs. UVB irradiation for experimental concentrations.

The ATP bioluminescent assay (PROMEGA) is a reliable method to determine cell viability by quantifying ATP. In the HaCaT cells, the ATP content was significantly (*p* < 0.05) increased in cells pre-treated for 4 h with the lowest concentration (10 µg/mL) of the tested samples when compared to untreated cells independent of UVB exposure (Figure 5A,C,E,G). However, after 24 h of pre-exposure to the tested samples, only compounds **1** (Figure 5E) and **3** (Figure 5G) were able to maintain ATP above control cell levels in UVB irradiated cells. Intriguingly, compound **3** was able to increase ATP in UVB irradiated cells to above levels observed in non-irradiated cells at the 100 µg/mL concentration (Figure 5G).

The tested samples were less active in the SK-MEL-1 cells when pre-treated for 4 h as evidenced by depleted ATP levels in UVB irradiated cells when compared to non-irradiated cells (Figure 5B,D,F,H). In contrast, 24-h pre-treatment resulted in all tested samples increasing ATP levels in UVB-irradiated cells when compared to cells not pre-treated with the tested samples (Figure 5B,D,F,H). The closer analysis also revealed that compounds **2** and **3** at 10 µg/mL were able to significantly increase ATP to above levels observed in non-UVB irradiated cells (Figure 5D,H).

### 2.5. The Tested Samples Inhibited Caspase 3 Activation and Was Not Cytotoxic in UVB Irradiated Skin Cells

To confirm the anti-apoptotic potential of the tested samples the activation of caspases 3/7 was investigated as these are the causes of cell death via apoptosis.

**Figure 6:**
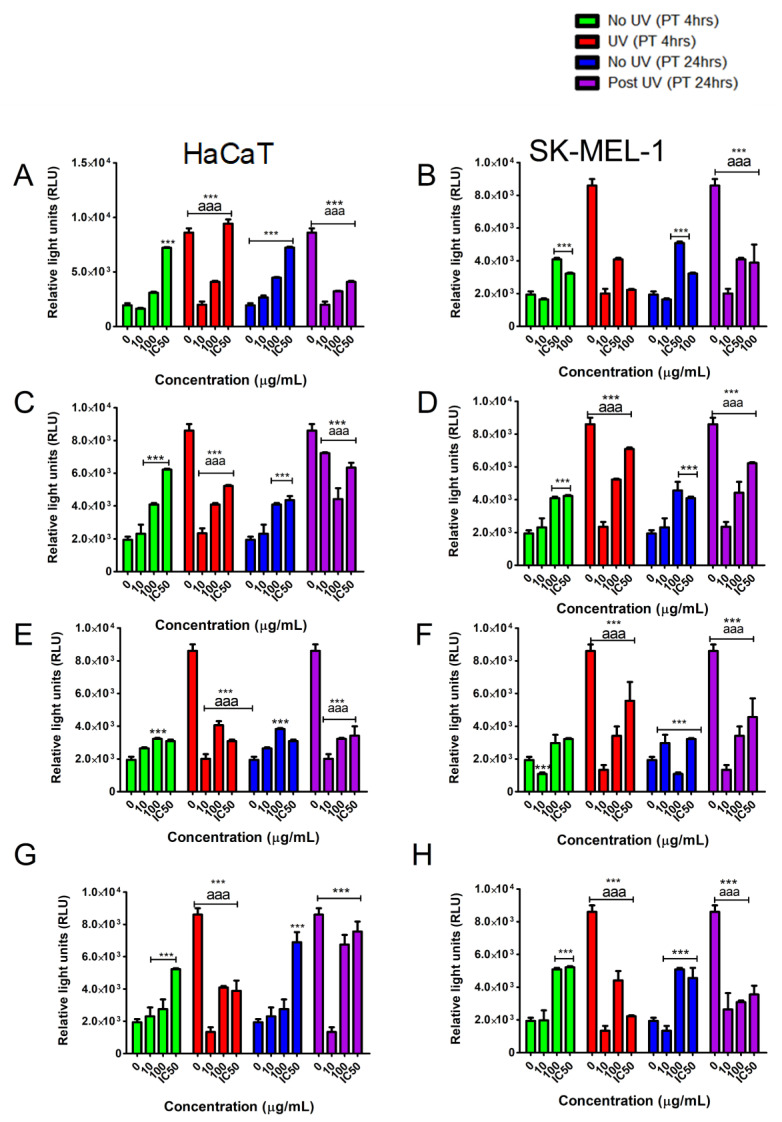
Pre-treatment with tested samples [TE (**A**,**B**); compound **1** (**C**,**D**); compound **2** (**E**,**F**); compound **3** (**G**,**H**)] effects in caspase 3 activation in skin cells exposed to UVB irradiation. Vertical lines indicate standard deviation. *** *p* < 0.001 control vs. experimental concentrations; ^aaa^ *p* < 0.001 No UVB irradiation vs. UVB irradiation for experimental concentrations.

All tested samples were able to reduce caspase activation in UVB irradiated cells as shown in Figure 6, suggesting a protective action against the UV-induced cell death via apoptosis. We observed that the lower test concentrations were more effective in significantly reducing apoptosis of UVB irradiated cells. We also show that the reduced apoptotic effects are dependent on the induction of cell damage by UVB exposure as the non-irradiated cells appeared to have a dose-dependent increase in caspase activity and resultant apoptotic cell death.

**Figure 7:**
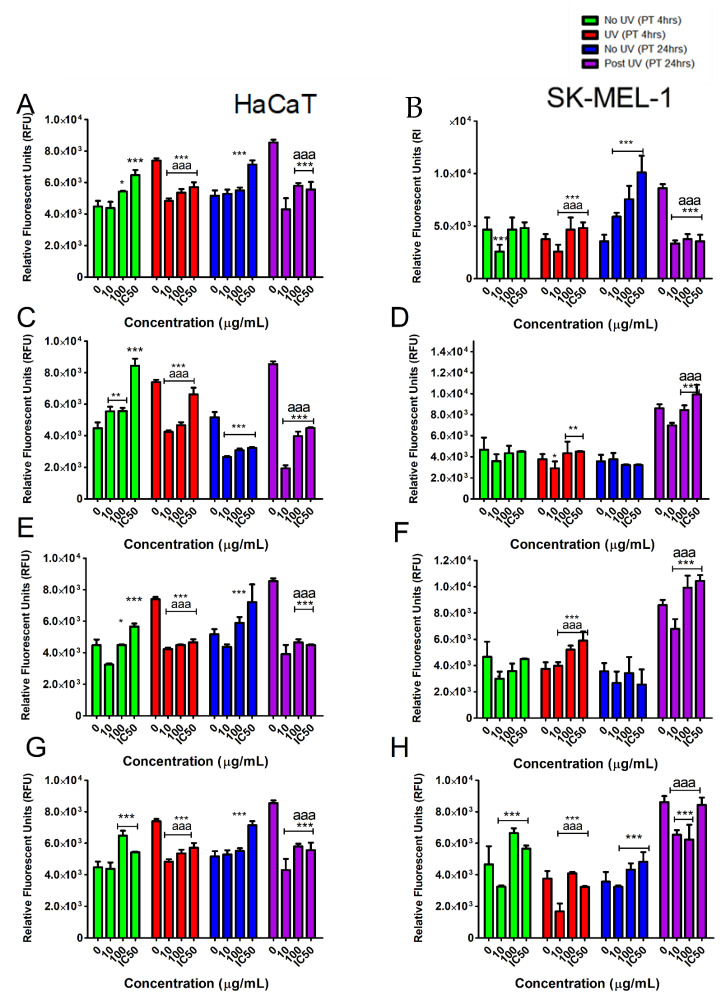
Pre-treatment with tested samples [TE (**A**,**B**); compound **1** (**C**,**D**); compound **2** (**E**,**F**); compound **3** (**G**,**H**)] and their cytotoxic potential in skin cells exposed to UVB irradiation. Vertical lines indicate standard deviation. * *p* < 0.05, ** *p* < 0.01, *** *p* < 0.001 control vs. experimental concentrations; ^aaa^ *p* < 0.001 No UVB irradiation vs. UVB irradiation for experimental concentrations.

Further evaluation of the cytotoxic potential of the test compounds revealed toxicity only at the highest concentrations tested in both cell lines. This was indicated by elevated fluorescence detected in dead cells (Figure 7). In combination with UVB irradiation, the tested samples induced marked decrease to cytotoxicity at low concentrations. Thus, the low concentrations of tested samples appear to have a photo-protective ability as they ameliorate UVB induced cell damage.

### 2.6. Modulation of UVB-Induced Oxidative Damage by the Tested Samples

Exposure to UVB light is known to induce oxidative stress and subsequent lipid peroxidation [31]. We, therefore, measured the levels of malondialdehyde (MDA) as a biomarker for lipid peroxidation.

**Figure 8:**
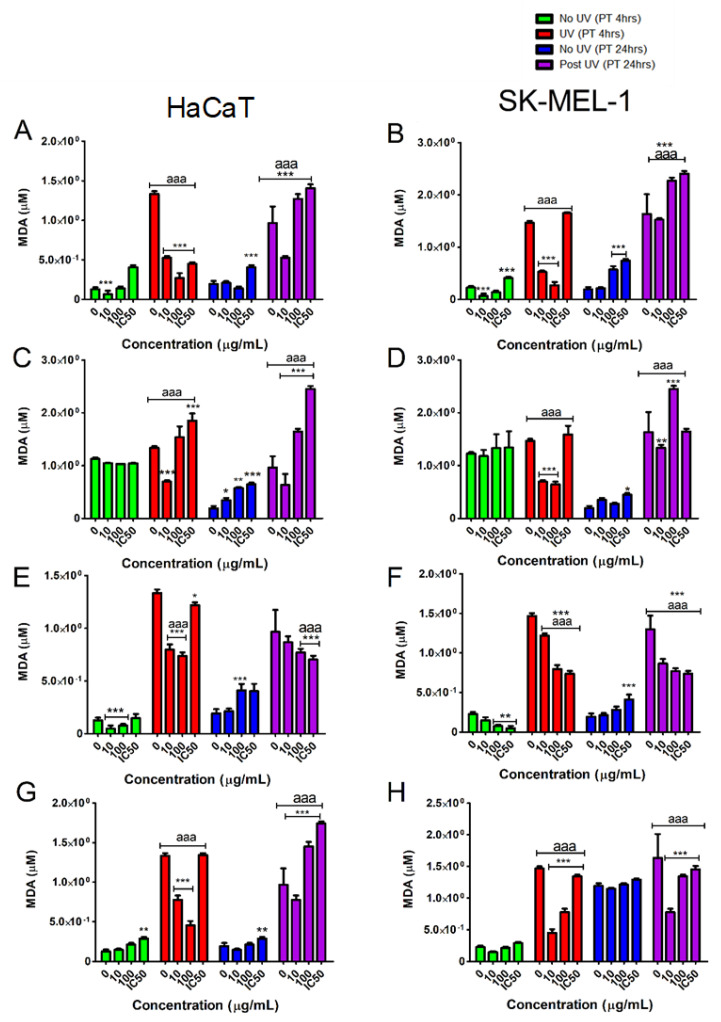
Protective effects of the tested samples [TE (**A**,**B**); compound **1** (**C**,**D**); compound **2** (**E**,**F**); compound **3** (**G**,**H**)] against lipid peroxidation after 4- and 24-h treatment before exposure to UVB irradiation. Vertical lines indicate standard deviation. * *p* < 0.05, ** *p* < 0.01, *** *p* < 0.001 control vs. experimental concentrations; ^aaa^ *p* < 0.001 No UVB irradiation vs. UVB irradiation for experimental concentrations.

From our investigation, it became clear that after a 4-h pre-treatment with the tested samples, both cell lines exposed to UVB displayed an overall hormetic response in which lower concentrations (10 and 100 µg/mL) significantly (*p* < 0.05) decreased the levels of MDA to below those observed in cells with no isolate intervention (Figure 8A–H). An exception to this was observed in HaCaT pre-exposed to compound **1**, where only the lowest concentration reduced MDA significantly (*p* < 0.05). We further showed that compound **1** at the lowest concentration was able to circumvent MDA formation by UVB to below levels observed in cells not exposed to UVB. This highlights compound **1** as the most protective compound.

In contrast, 24-h pre-treatment with the tested samples appeared to have a pro-oxidant effect as we show a significant (*p* < 0.05) increase in MDA levels following UVB exposure in the HaCaT and SK-MEL-1 cell lines with only the lowest concentration (10 µg/mL) offering protection (Figure 5A–H). However, compound **2** showed a significant (*p* < 0.05) reduction in MDA levels at all tested concentrations in both cell lines after UVB exposure (Figure 6C,D).

### 2.7. The Effect of Tested Samples on GSH Levels in Skin Cells Exposed to UVB

**Figure 9:**
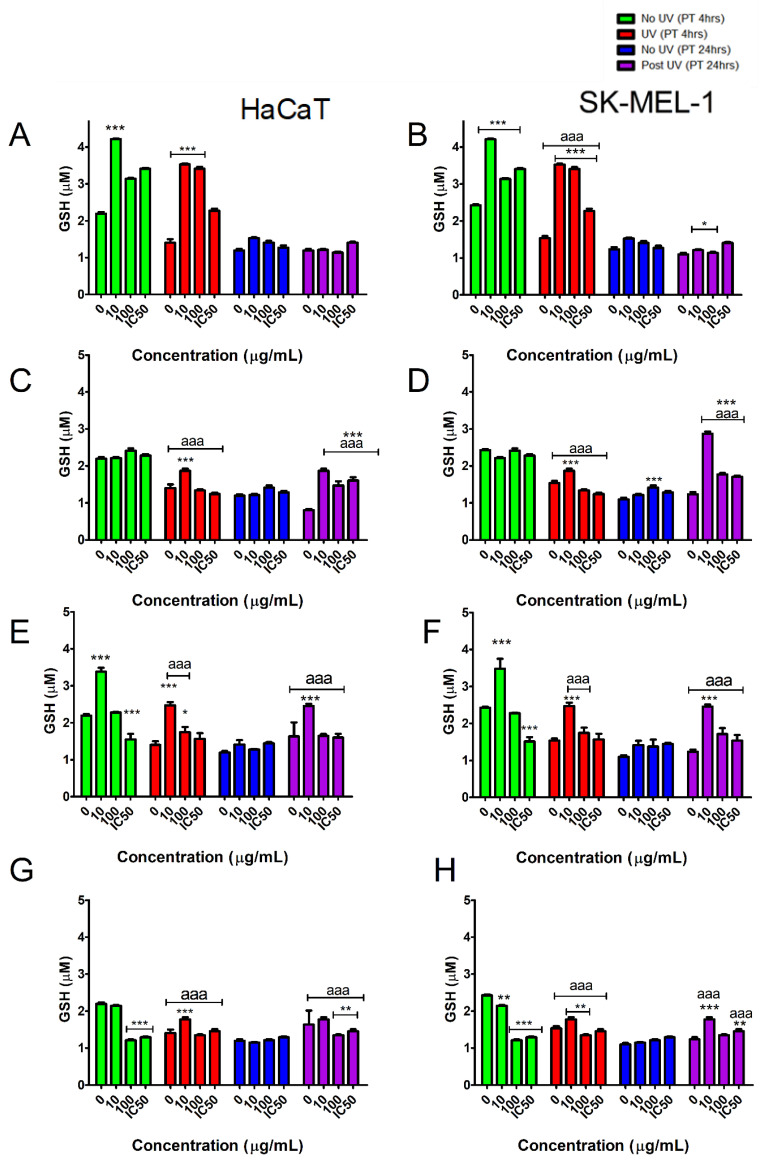
Effect of tested samples [TE (**A**,**B**); compound **1** (**C**,**D**); compound **2** (**E**,**F**); compound **3** (**G**,**H**)] on glutathione (GSH) concentration in skin cells exposed to UVB irradiation. Vertical lines indicate standard deviation. * *p* < 0.05, ** *p* < 0.01, *** *p* < 0.001 control vs. experimental concentrations; ^aaa^ *p* < 0.001 No UVB irradiation vs. UVB irradiation for experimental concentrations.

Changes in GSH levels are important when assessing cell defense responses and are an indicator of oxidative stress. The GSH content declined immediately after exposure to UVB in all tested cell models (Figure 9) when no intervention with tested samples was applied. Pre-treatment with TE for 4 h appeared to significantly (*p* < 0.05) improve the GSH content in both UVB exposed and non-exposed cells. However, 24 h pre-treatment with TE did not improve GSH levels. Compound **2** was only active at the lowest concentration tested in HaCaT cells when pre-treated for 4 h and 24 h. In contrast, SKMEL-1 pre-treated cells showed better resistance profiles to UVB irradiation as evidenced by improved GSH levels. A similar pattern was observed for both compounds **1** and **3**.

Closer scrutiny of the data showed that the cells pre-treated with compounds **1‒3** had higher GSH levels after UVB exposure when compared to cells not exposed to UVB. This suggests that GSH synthesis is boosted in cell stress systems by the tested samples.

## 3. Discussion

UVB irradiation is seen as the primary extrinsic contributor to damage of the epidermal layer of skin through mechanisms associated with oxidative distress in cells [1]. This is associated with the potential of UVB to disrupt vital enzymatic and non-enzymatic antioxidant systems in cells as well as the execution of apoptosis [2,3] and makes it important to explore novel and effective antioxidant molecules to protect cells from UVB-induced damage [2].

Previous studies have reported that rooibos total extracts markedly enhanced UVB-induced inhibition of cell viability, proliferation, and induction of apoptosis in a UVB/Keratinocyte model [22,32]. However, the potential of compound **2** and its derivatives to attenuate oxidative stress in UVB/keratinocyte models is currently unclear. Its application in skincare is made appealing by its ability to cross physiological barriers [27] while still maintaining pharmacological activity in skin cells [22,32].

To systemically evaluate the cytoprotective effects of compound **2** and derivatives, we measured cell viability in the UVB/Keratinocyte (HaCaT) a pre-malignant cell line and UVB/Melanocyte (SK-MEL-1), originating from tumourigenic cells, models that were pre-exposed to the tested samples for 4 and 24 h. From our study, **1** was the most active in preventing the loss of cell viability after exposure to UVB compared to all other tested samples in the UVB/keratinocyte model, we observed that this protective effect was most evident when cells were pre-treated for 4 h.

This suggests that compound **1** is likely to target and elicit an early defence response against UVB damage. This is in contrast to the UVB/melanocyte model which showed a 24-h pretreatment with compound **2** to maintain viability which is not a desirable effect when dealing with tumorigenic cells. This suggests that the effects of the tested samples are both time and cell type-dependent. While this is the first study to evaluate the protective effects of compound **2** in a UVB toxicity model it is important to note that this data is in contrast to previous studies which elaborate on the synergism of rooibos polyphenolic constituents over rooibos monomeric compounds [22,32,33].

The MTT assay used to evaluate cell viability is based on the ability of metabolically active cells to metabolise the salt into a soluble formazan product [34]. To verify that the tested samples maintained metabolic activity after UVB exposure we assessed intracellular ATP levels. UVB exposure in the models significantly depleted ATP in cells not pre-treated with tested samples, this may be attributed to UVB directly inhibiting ATP synthesis in the skin [35]. Alternatively, the reduction in cellular ATP seen after irradiation may also reflect increased ATP usage by cells recovering from UV damage [36]. Our data indicate that a 4-h pretreatment with 10 µg/mL of the tested samples was sufficient to elevate ATP levels in cells exposed to UVB. This indicates that at the lowest tested concentration, TE along with compound **2** stimulates ATP production in both models. The higher levels of ATP may contribute to cell survival by supporting energy-dependent DNA repair processes [36] as well as stimulating pigmentation processes in keratinocytes to directly protect against UV [37]. These findings underscore the potential of the tested samples in reducing the genotoxic effects of UV to maintain genomic stability and to prevent malignant transformation.

It is important to note that at the highest concentrations tested, the experimental samples decreased ATP levels in UVB irradiated and non UVB irradiated cells, suggesting that these compounds have a modulatory role on cell energy pathways that is concentration-dependent. Functional mitochondria and the NAD:NADH ratio are essential to maintaining cellular ATP levels. Strong evidence has found that polyphenols present in total rooibos extracts may inhibit the mitochondrial respiratory chain [38] and may explain the loss of ATP at higher concentrations. This observation is in line with previous studies that highlight the therapeutic potential of rooibos extracts in skin cancer [22,32,38].

UVB irradiation is well known to cause skin damage and premature ageing through the excessive production of ROS and induction of oxidative stress [2]. In this study, the suppression of MDA generation indicated that certain concentrations of dihydrochalcone of *A. linearis* can decrease skin damage and protect skin cells from the oxidative stress induced by UVB irradiation. Similarly, previous studies on rooibos and its polyphenols have been shown to possess antioxidant potential. Several mechanisms have been proposed, including direct scavenging of ROS as well as up-regulation of ROS detoxifying enzymes [20,21,33]. Our work demonstrates that compound **2** and its derivatives have a favourable biological effect within a zone of low dose pre-treatment and UVB stress while higher concentrations, above a threshold of 100 µg/mL elicits a high degree of oxidative damage as ROS levels exceed the endogenous antioxidant capacity. The potential of rooibos and its constituents to enhance the Fenton reaction has been established [33] and associated with the metal-chelating properties of rooibos and its polyphenols [32] may contribute to the pro-oxidant effects observed.

Several lines of evidence have indicated that the antioxidant functionality of skin is modulated by the redox status of GSH under UVB stress to maintain homeostasis [39]. The epidermis contains glutathione (GSH), a hydrophilic tripeptide (l-γ-glutamyl-l-cysteinylglycine), with characteristic sulfhydryl groups readily reacting with ROS and other mediators of oxidative stress. Since glutathione, alone or as a co-factor of several enzymes (GSH peroxidases, reductases, and GSH-*S* transferase), plays a pivotal role in the biological antioxidant defence systems, it was chosen as the most relevant parameter for the quantification of the UVB/keratinocyte (HaCaT) and UVB/melanocyte (SK-MEL-1) redox status. Reduced GSH is naturally present in the skin and its intracellular levels are regulated by several metabolic pathways. Reduced GSH forms oxidised GSH after detoxification of ROS and must be converted to reduced GSH before detoxification of ROS can continue. Therefore, reduced GSH is indicative of the redox status of skin cells and the effectiveness of regenerating systems [39]. Our results showed that the total rooibos extract was biologically more active in inducing an increase in GSH than compound **2** when keratinocytes and melanocytes were exposed to UVB especially at the lowest concentration tested, however, compound **2** and its derivatives were still able to significantly rescue the GSH status in exposed cells. These results indicate that compound **2** and its derivatives may have a role to play in GSH recycling or enhancing GSH production in skin cells exposed to UVB. This suggests a protective, antioxidant effect against UVB damage at low concentrations.

Growth inhibition and apoptosis by rooibos is well documented for pre-malignant and malignant skin cells suggesting its potential as a potent drug candidate against hyperproliferative diseases [20,22,34,40]. However, little work has been done to establish the protective effects of rooibos and its polyphenols against UVB-induced apoptosis. Our work aims to address this knowledge gap. The results of our study clearly indicate that pre-treatment with total extract, compounds **1**, **2**, and **3** inhibited the activation of executioner caspases in a dose-dependent manner with lower concentrations showing the most potent inactivation. We further show that in agreement with previous studies apoptosis was enhanced by all tested samples when UVB was not used as a stressor [22,32,38]. This suggests that the anti-apoptotic effects are UVB dependent and that the tested samples do not act as a photosensitiser to apoptosis. The tested samples were able to induce photoprotective effects against UVB induced cytoxicity at low concentrations in both skin cell models while higher concentrations induced cytotoxicity. The cytotoxic effects of rooibos extracts have been well established in studies using cancer models [20,22]. Our study shows that the potential of lower concentrations of rooibos extracts show promise as cytoprotective agents.

Apoptosis and the sustained activation of caspase 3 is an energy-dependent process [40]. The decreased caspase activity may be associated with inhibition of ATP producing pathways since UVB irradiation is known to target the mitochondria [41] and inhibit glycolysis due to elevated ROS [42]. The mitochondrial succinate dehydrogenase enzyme activity can be indirectly detected using the MTT assay. Pre-treatment with high concentrations of test compounds before UVB-irradiation resulted in a significant decrease of MTT reduction, indicating a decrease of metabolically active cells compared to non-UVB control. Pre-treatment with low concentrations protected the metabolic activity of cells, as reflected by high conversion of MTT, suggesting that both malignant and premalignant cell types used in this study are susceptible to cell death through pathways that are independent of apoptosis. This is supported by data showing elevated ROS damage biomarkers.

From the structural-activity relationships of the isolated compounds, it was noted from compounds **2** and **3** are structurally similar dihydrochalcones with a *C*-glucose at C-3′. However, they differ due to the presence/absence of the 3-OH of ring B (Figure 1). On the other hand, compounds **1** and **2** are closely related except for the sugar unit, the presence of deoxysugar and the extra ether linkage decrease the polarity of the parent compound **2** and may be one reason for the observed differences in activities. This structural-activity relationship of the compounds **1**, **2** and **3** is suggestive of the observed activity order of **1** > **2** > **3** in all treated cells at 4 h. Keeping these similarities and slight differences among the derivatives in mind, we hypothesised for the observed similar or time-dependent activities of the total extract, **1**, **2** and **3** on skin cells irradiated with UVB irradiation.

The excessive ROS levels associated with UVB exposure downregulates anti-apoptotic proteins and upregulates pro-apoptotic proteins, resulting in interference with the normal release of cytochrome-c, which activates the caspase family proteins, and finally induces cell death [2,5]. The alterations in UV-induced apoptosis regulation may have an important impact on the induction of skin photoaging and skin cancer. Previous studies have demonstrated that the inhibition of excessive apoptosis and the accelerated elimination of ROS induced by UVB could protect the skin from UVB-induced ageing and carcinogenesis [43]. Our results showed that the tested compounds effectively attenuated UVB-induced oxidative stress and apoptosis, thereby protecting the skin from UVB-induced damage. It must be noted that an attenuated apoptotic response to stress may not be desirable in malignant cells, however, the potential of the test compounds to still induce cell death by other mechanisms must be further investigated. The pre-treatment regimen employed by this study suggests that the protective effects of tested samples involve some degree of cellular adaptation, resulting in enhanced protection against UVB damage.

## 4. Materials and Methods

### 4.1. Plant Material

Freshly dried and pulverized green rooibos plant material was generously donated by the Rooibos Ltd. (Western Cape, South Africa) in April 2019. The plant was stored at room temperature and in a sealed container protected from light before use.

### 4.2. Chemicals and Equipment

Analytical/HPLC grade chemicals supplied by Sigma-Aldrich (Cape Town, South Africa) and Merck (Cape Town, South Africa) were used in this study. Organic solvents and acids such as dichloromethane (DCM), ethyl acetate, acetone, methanol (HPLC grade), ethanol, formic acid, and acetic acid were supplied by Merck. Thin-layer chromatography (TLC) plates of Silica gel 60 PF_254_ pre-coated on aluminium, Silica gel 60 H (0.040–0.063 mm particle size, from Merck and Sephadex LH-20 (Sigma-Aldrich) were used. De-ionized water was generated in-house using a Merck Millipore (18.2 MΩ·cm at 25 °C) water purification system. Cell culture reagents (Section 4.4, Section 4.5, Section 4.6, Section 4.7 and Section 4.8) were supplied by ThermoFisher (Cape Town, South Africa) unless otherwise stated.

Chromatographic fingerprints and final purification of compounds were conducted on a reverse-phase high-performance liquid chromatography system (Shimadzu, Kyoto, Japan) coupled with a photo-diode array detector and equipped with a Discovery^®^ C_18_ column (25.0 × 1.0 cm, SUPELCO, St. Louis, MO, USA). Structural information of compounds was obtained using nuclear magnetic resonance (NMR) on an Ascent 400 MHz NMR spectrometer (Bruker, Rheinstetten, Germany) in acetone-*d_6_* and dimethyl sulfoxide-*d_6_*. HRMS analysis was conducted on an Ultimate 3000 LC system (Dionex, Sunnyvale, CA, USA) coupled to a Bruker QTOF with an electrospray ionization (ESI) interface working in the positive ion mode. UV measurements were done with a SPECTROstar Nano 2450 UV-vis spectrophotometer (BMG LABTECH, Ortenberg, Germany). IR spectra were recorded on a Perkin Elmer Fourier-Transform Infrared Spectrometer 2000, equipped with a PerkinElmer Spectrum 100 universal ATR-FTIR accessory (Perkin Elmer, Llantrisant, Wales, UK) at transmission mode of 400–4000 cm^−1^.

### 4.3. Extraction and Isolation of Linearthin, Aspalathin and Nothofagin

One kilogram of green *A. linearis* plant material was exhaustively extracted with acetone (4 L) in the dark for 24 h at room temperature and then filtered. After concentration, the total extract obtained (30 g, 3%), was then transferred to vials and kept dry till further use. The extract was later subjected to silica gel column (75 × 10 cm) using gradient elution system (1% MeOH/DCM to 100% MeOH) and 250 mL fractions were collected. The concentrated fractions were monitored using TLC and combined to produce twenty-two main fractions, labelled I–XXII.

Gravity column chromatography of the main polar fraction XI (3 g) on a silica gel column (40 × 8 cm, gradient mode) using EtOAc, acetone and MeOH afforded a semi-pure compound, 1.2 g), which was further purified on Sephadex column (using gradient mode; 100% H_2_O to 100% MeOH) to yield the major compound aspalathin (**2**, 0.7 g). The same compound was also isolated from the main fractions VIII, X, and XII. The main fraction XV (0.5 g) was subjected to the silica gel and then Sephadex column chromatography to yield nothofagin (**3**, 64 mg). Sequential processing of the main fraction XXII (1 g) on a silica gel column afforded three subfractions; A (382 mg), B (207 mg), and C (124 mg). Subfractions A and B were identified as the same as compound **2** from TLC and semi-prep HPLC showing similar retention times/factors. The subfraction C was applied to a Sephadex column and further purified with HPLC using 80% aqueous acetonitrile in isocratic mode resulted in a novel compound linearthin (**1**, 10 mg, R_t_; 30 min) as an amorphous white-yellowish powder. UV λ_max_ 283.6 and 332 nm (MeOH). FTIR: 1050, 1705, 2950 and 3340 cm^−1^. HRMS *m*/*z* 443.1146 [M-H]^+^; 311.0, 269.0, 233.0, 221.0, 179.0, 167.0. ${\left[\alpha \right]}_{25}^{D}$: –13.3 (*c*: 0.04; MeOH). ^1^H- and ^13^C-NMR see Table 1. To prepare the acetate derivative **1a**, 5 mg of **1** was mixed with pyridine and acetic anhydride (2.0 mL each; 1:1). The resulting mixture was kept at 37 °C for 24 h and then partitioned in ethyl acetate, washed thrice with water and finally purified on silica gel column which yielded 2 mg of compound **1a**. ^1^H- and ^13^C-NMR see Table 1.

### 4.4. Cell Culture and Cell Conditioning

The human epidermal keratinocytes (HaCaT) and melanoma (SK-MEL-1) cells used in this study were cultured in Roswell Park Memorial Institute (RPMI) 1640 media supplemented with antibiotics (100 U/mL streptomycin, 100 U/mL penicillin) and fetal calf serum (FCS, 10%). Cells were maintained at 37 °C in a humidified atmosphere of 5% CO_2_.

For experiments, before exposure to UVB, the cells were conditioned with various concentrations of total extract and isolated compounds thereof for 4 or 24 h. These periods were selected based on the half-life of the compounds as well as previous studies [22,32]. Exposure to UVB was done according to a previous study. Cells were seeded (3 × 10^4^) in 96-well tissue culture microtitre plates. Cell culture media was aspirated and the cells were overlayed with PBS. The cells were exposed to UVB light (80 mJ/cm^2^) without the plastic lid. A UVIlink UV Crosslinker (UVitek Ltd., Aberdeen, UK) was fitted with six 8 Watt UV tubes giving a wavelength of 302 nm (Vilber Lourmat, Collégien, France) [22]. Control plates were not exposed to UVB light.

### 4.5. MTT Cytotoxicity Assay

The MTT assay has been extensively used for determining the cytoprotective effects of several phytochemicals [44]. To monitor cell viability as a measure of metabolic activity the MTT assay was performed. The conditioned HaCat and SK-MEL-1 cells (15,000 cells per well in 96-well plates) were incubated for 2 h using 100 μL MTT solution (5 mg/mL) after PBS washing. The MTT formazan crystals were then solubilised by DMSO resuspension. The optical density of the formazan product was read at 570 nm wavelength with a reference wavelength of 690 nm. The half-maximal inhibitory concentration (IC_50_) was determined.

### 4.6. Cell Viability—ATP Assay

The ATP bioluminescent assay was used to quantify cellular ATP. The Cell Titer-Glo^®^ assay (Promega, Madison, WI, USA) was carried out according to the manufacturer’s instructions. Cells were seeded in a white microtitre plate (20,000 cells per well) to which the ATP Cell Titer-Glo^®^ Reagent was added (50 μL). The plate was incubated for 30 min at room temperature (RT) in the dark. Luminescence was measured on a Multiskan Spectrum plate reader (Thermo Fisher Scientific, Waltham, MA, USA). Luminescence is proportional to ATP levels and expressed as relative light units (RLU).

### 4.7. Viability, Cytotoxicity, and Apoptosis

The ApoTox-Glo™ Triplex Assay (Promega) was used to assess cytotoxicity and apoptosis events in the same cell-based assay well according to the manufacturer’s instructions with no deviation from standard protocol. Both luminescence and fluorescence were detected using a plate reader.

### 4.8. Antioxidant Response

The thiobarbituric acid reactive substances (TBARS) was used to quantify the levels of melondialdehyde (a marker of lipid peroxidation) in the supernatant of UVB irradiated and non-irradiated cells. The experiments were conducted as previously described [45]. The optical density was measured at 532 nm with a reference wavelength of 600 nm using a plate reader. The mean optical density of five samples per treatment was calculated and divided by the absorption coefficient (156 mM^−1^). Results were expressed as MDA concentration (μM). Reduced glutathione (GSH) was quantified using an in-house protocol with minor modifications [21]. After the addition of reagents, the cells were lysed to allow quantification of GSH colourimetrically using a plate reader.

### 4.9. Statistical Analyses

Biological experiments were conducted in triplicate (independently). Statistical data were analysed by one way ANOVA and the Bonferroni test for multiple group comparisons unless otherwise stated. The software used for all analyses was GraphPad Prism v. 5.0 software (GraphPad Software Inc., San Diego, CA, USA). Results were considered statistically significant when *p* < 0.05.

## 5. Conclusions

The findings of this study demonstrated for the first time the application of linearthin and other chalcone derivatives from rooibos to protect human-derived skin cells from excessive ROS generated by UVB exposure in vitro. The isolates used in this study can improve cell viability and inhibit apoptosis induced by UVB exposure by likely exploiting pathways associated with the established antioxidant properties of rooibos. We show that isolates reduced biomarkers associated with lipid oxidative damage and improved the oxidative status of the cell. Thus, linearthin and other chalcone derivatives can be beneficial in protecting against the harmful effects of solar UVB radiation and consequent oxidative stress in humans. The results of this work further warrants the need for studies to further establish them as beneficial agents in the prevention of UV-induced immune suppression. In addition, the isolation of linearthin (**1**) as a new structure from *A. linearis*, may represent a biosynthetic intermediate to the synthesis aspalathin from phloretin which will need more investigation.

## Figures and Tables

**Table 1:** ^1^H- and ^13^C-NMR data for compounds **1**–**3**.

Position	Linearthin (1) (DMSO-*d*_6_)		Linearthin Acetate (1a) (CDCl_3_)		Aspalathin (2) (DMSO-*d*_6_)		Nothofagin (3) (DMSO-*d*_6_)	
	δ_C_	δ_H_ (*J*, Hz)	δ_C_	δ_H_ (*J*, Hz)	δ_C_	δ_H_ (*J*, Hz)	δ_C_	δ_H_ (*J*, Hz)
1	132.4		139.2		132.9		132.1	
2	116.3	6.61 (*d*, 1.7)	123.4	7.04 ^†^	116.2	6.61 (*br s*)	129.6	7.09 (*d*, 8.1)
3	143.6		141.8		145.4		115.5	6.73 (*d*, 8.1)
4	145.4		141.8		143.7		155.8	
5	115.9	6.63 (*d*, 8.0)	123.4	7.06^†^	115.9	6.47 (*d*, 8.0)	115.5	6.73 (*d*, 8.1)
6	119.1	6.49 (*br d*, 8.0)	126.5	7.09 ^†^	119.4	6.63 (*br d*, 8.0)	129.6	7.09 (*d*, 8.1)
1′	100.7		113.9		104.4		104.4	
2′	161.2		157.6		162.1		165.5	
3′	103.0		115.9		104.1		104.1	
4′	161.4		149.3		164.1		164.4	
5′	95.9	5.82 (*s*)	110.4	6.45 (*s*)	95.0	5.95 (*s*)	95.0	5.99 (*s*)
6′	164.6		149.4		165.3		162.4	
α	43.6t	3.18 (*t*, 7.6) 3.35 *	45.0	3.20 (2H, *t*, 7.0)	45.9	3.32 (2H, *m*)	46.0	3.28 (2H, *t*, 8.7)
β	29.1	2.75 (2H, *t*, 7.6)	28.9	2.96, 2.94 (*t*/each, 7.0)	30.1	2.83 (2H, *t*, 7.9)	30.1	2.82 (2H, *t*, 7.8)
CO	203.3		196.9		205.0		204.8	
1″	33.3	3.10, 2.91 (*d*/each, 16.4)	35.0	3.15, 3.29 (*d*/each, 16.8)	74.0	4.52 (*d*, 9.9)	74.3	4.58 (*d*, 9.8)
2″	117.8		116.2		70.9	3.89 (*t*, 8.7)	71.2	3.94 (*t*, 8.7)
3″	80.0	3.88 (*d*, 8.0)	77.8	5.47 (*d*, 7.0)	79.3	3.22 (*t*, 7.6)	79.4	3.22 *
4″	74. 1	3.83 (*br d*, 7.5)	77.8	5.27 (*br t*, 6.2)	71.1	3.14 (*br t*, 7.3)	70.8	3.18 *
5″	84.2	3.77 (*m*)	79.5	4.25 ^††^	81.7	3.18 *	81.7	3.19 *
6″	63.8	3.43 * 3.54 (*br d*, 12.5)	64.5	4.27 ^††^ 4.03 (*br d*, 10.6)	61.7	3.42 (*dd*, 3.6, 11.6) 3.66 (*d*, 11.6)	61.7	3.48 (*br d*, 11.5) 3.70 (*d*, 11.5)

*, ^†^, ^††^ overlapped signals in the same column.

## Data Availability

Not applicable.
